# Exploration and Preliminary Investigation of Wiled *Tinospora crispa*: A Medicinal Plant with Promising Anti-Inflammatory and Antioxidant Properties

**DOI:** 10.3390/cimb48010070

**Published:** 2026-01-09

**Authors:** Salma Saddeek

**Affiliations:** Department of Chemistry, Faculty of Sciences, University of Hafr Al Batin, Hafr Al Batin 39524, Saudi Arabia; salmayms@uhb.edu.sa

**Keywords:** *Tinospora crispa* (L.) Hook.f. & Thomson, phytochemical profiling, chemotype variation, anti-inflammatory activity, SNP analysis

## Abstract

Background and Rationale: *Tinospora crispa* (L.) Hook.f. & Thomson (*T. crispa)* is a climbing medicinal plant with long-standing ethnopharmacological use, particularly in inflammatory and hepatic disorders and cancer-related conditions. There is a knowledge gap regarding how wild versus cultivated ecotypes differ in chemotype, bioactivity, and safety, and how this might support or refine traditional use. Study Objectives: This study aimed to compare wild and cultivated ecotypes of *T. crispa* from the Nile Delta (Egypt) in terms of quantitative and qualitative phytochemical profiles; selected in vitro biological activities (especially antioxidant and cytotoxic actions); genetic markers potentially associated with metabolic variation; and short-term oral safety in an animal model. Core Methodology: Standardized extraction of plant material from wild and cultivated ecotypes. Determination of total phenolics, total flavonoids, and major phytochemical classes (alkaloids, tannins, terpenoids). Metabolomic characterization using UHPLC-ESI-QTOF-MS, supported by NMR, to confirm key compounds such as berberine, palmatine, chlorogenic acid, rutin, and borapetoside C. In vitro bioassays including: Antioxidant activity (e.g., radical-scavenging assay with EC_50_ determination). Cytotoxicity against human cancer cell lines, with emphasis on HepG2 hepatoma cells and calculation of IC_50_ values. Targeted genetic analysis to detect single-nucleotide polymorphisms (SNPs) in the gen1 locus that differentiate ecotypes. A 14-day oral toxicity study in rats, assessing liver and kidney function markers and performing histopathology of liver and kidney tissues. Principal Results: The wild ecotype showed a 43–65% increase in total flavonoid and polyphenol content compared with the cultivated ecotype, as well as substantially higher levels of key alkaloids, particularly berberine (around 12.5 ± 0.8 mg/g), along with elevated chlorogenic acid and borapetoside C. UHPLC-MS and NMR analyses confirmed the identity of the main bioactive constituents and defined a distinct chemical fingerprint for the wild chemotype. Bioassays demonstrated stronger antioxidant activity of the wild extract than the cultivated one and selective cytotoxicity of the wild extract against HepG2 cells (IC_50_ ≈ 85 µg/mL), being clearly more potent than extracts from cultivated plants. Genetic profiling detected a C → T SNP within the gen1 region that differentiates the wild ecotype and may be linked to altered biosynthetic regulation. The 14-day oral toxicity study (up to 600 mg/kg) revealed no evidence of hepatic or renal toxicity, with biochemical markers remaining within physiological limits and normal liver and kidney histology. Conclusions and Future Perspectives: The wild Nile-Delta ecotype of *T. crispa* appears to be a stress-adapted chemotype characterized by enriched levels of multiple bioactive metabolites, superior in vitro bioactivity, and an encouraging preliminary safety margin. These findings support further evaluation of wild *T. crispa* as a candidate source for standardized botanical preparations targeting oxidative stress-related and hepatic pathologies, while emphasizing the need for: More comprehensive in vivo efficacy studies. Cultivation strategies that deliberately maintain or mimic beneficial stress conditions to preserve phytochemical richness. Broader geographical and genetic sampling to assess how generalizable the present chemotypic and bioactivity patterns are across the species.

## 1. Introduction

Climbing species within the *Tinospora genus* (family *Menispermaceae*) constitute a pharmacologically important but under-investigated group of medicinal plants. Regionally grouped under the ethnobotanical label “Phyllo-stem arboreum”—which includes *T. crispa*, *T. cordifolia*, *Cissampelos pareira*, and *Sinomenium japonica*—these woody lianas have been traditionally employed across tropical Asia for the treatment of fever, inflammation, metabolic disorders, and microbial infections [1,2]. While these species share convergent morphological traits such as succulent stems and cordate leaves, they exhibit considerable inter- and intraspecific phytochemical variation due to environmental pressures and genetic drift [3,4].

Despite their long-standing therapeutic applications, comparative studies integrating their metabolomic profiles, pharmacological activities, and adaptive responses to ecological stressors remain limited [5,6]. To address this gap, a combined approach of genomic and chemical fingerprinting has become indispensable for authenticating and standardizing medicinal plants. DNA barcoding tools (e.g., ITS and matK regions) effectively resolve taxonomic ambiguity among closely related species—a vital step for preventing misidentification and adulteration within the “Phyllo-stem arboreum” group [7].

Parallel to this, analytical profiling using LC-MS/MS and NMR techniques enables precise quantification of pharmacologically relevant metabolites, facilitating direct correlations between compound abundance and biological activity [8,9]. In wild ecotypes such as *T. crispa*, this dual fingerprinting strategy also reveals how somatic mutations—such as the identified C → T SNP in the gen1 locus—may modulate biosynthetic pathways, leading to enhanced production of bioactive secondary metabolites under natural stress conditions [10,11]. These molecular and chemical markers thus serve not only as tools for authentication, but also as essential metrics for ensuring therapeutic consistency and industrial scalability [12,13].

Field investigations of wild *T. dactyloides* populations uncovered notable morphological deviations—such as leaf curling, stem lignification, and chlorophyll loss—compared to both herbarium specimens and cultivated references. These phenotypic adaptations are likely driven by environmentally induced somatic mutations (e.g., drought, UV radiation), which activate transcriptional programs that favor secondary metabolite biosynthesis for enhanced survival [14,15,16]. Interestingly, such morphological shifts often occur despite minimal genetic divergence (>98% ITS/matK sequence homology with reference specimens), supporting the hypothesis that these represent acclimatized chemotypes rather than new species [17,18].

This mismatch between genotype and phenotype underscores the potential of wild populations as reservoirs of superior bioactivity—although the mechanistic links between environmental pressures, genetic change, and therapeutic efficacy remain insufficiently explored [19]. Initial metabolomic screening confirmed *T. crispa* as the most chemically enriched species among the studied “Phyllo-stem arboreum” group, exhibiting particularly good levels of alkaloids and phenolic acids [20].

Wild-collected samples further demonstrated a 43–65% increase in flavonoids, tannins, and chlorogenic acid derivatives compared to cultivated counterparts—reflecting a metabolic shift toward enhanced antioxidative capacity under environmental stress [21,22]. Advanced metabolite profiling using UHPLC-ESI-QTOF-MS and nuclear magnetic resonance (NMR) spectroscopy confirmed the elevated presence of key bioactive constituents, such as cytotoxic protoberberines, anti-inflammatory phenolics, and hepatoprotective diterpenes, thereby corroborating the plant’s ethnopharmacological uses [23,24]. Contemporary research (2023–2025) has deepened our understanding of this phenomenon; advanced metabolomic studies have been instrumental in defining stress-induced metabolic signatures in *Tinospora* species [25], while recent SNP-based genotyping studies have successfully linked genetic markers with both chemotypic diversity and the enhanced production of bioactive compounds [26,27]. These modern, integrated approaches underscore the necessity of multimodal characterization to fully harness the therapeutic potential of stress-acclimatized chemotypes [28].

## 2. Materials and Methods

### 2.1. Plant Collection and Authentication

Wild and cultivated specimens of *T. crispa* were collected during peak growth (April–May 2024) from two contrasting agro-ecological zones in Egypt’s Nile Delta:

Wild ecotype: Specimens were harvested from stress-prone natural habitats characterized by sandy, nutrient-depleted soils, full sun exposure, and minimal irrigation (GPS coordinates: 30° N, 31° E).

Cultivated ecotype: Plants were greenhouse-grown under optimized conditions (loamy soil, 25–30 °C, daily irrigation, pH 6.8) at the Agricultural Research Center (ARC), Giza, Egypt.

Authentication and Voucher Deposition: The taxonomic identity of all collected plant material was confirmed by morphological comparison with a verified reference specimen of *T. crispa* (Family: *Menispermaceae*) deposited at the Cairo University Herbarium. The reference specimen (Collector: Ahmed El-Sayed, Collection Date: 15 March 2021) originated from the Cairo University Botanic Garden, Giza, Egypt. The accepted botanical name *T. crispa* was validated against the Plant of the World Online (POWO, Kew Science) database, with historical synonyms including Cocculus verrucosus (Roxb.) Wall. Representative voucher specimens from this study (Accession Nos. TC-EG-2024-01 to TC-EG-2024-12) were deposited at the National Herbarium of Egypt (CAIRC) for permanent archival and future reference.

### 2.2. Extract Preparation for Bioassays

Sample Preparation: Dried aerial parts (stems and leaves) of wild and cultivated *T. crispa* were ground to a fine powder using an electric mill. For each ecotype, three independent biological replicates (batches) were processed separately. This triplicate biological replication strategy was implemented to account for natural variability within plant populations and to ensure statistical robustness. The sample size (*n* = 3) was determined based on standard practices in phytochemical research and previous studies demonstrating that three replicates provide sufficient power to detect significant differences in secondary metabolite content with acceptable statistical confidence.

Extraction Protocol: Bioactive compounds were extracted using methanol as the solvent. Briefly, 50 g of plant powder was mixed with 500 mL of analytical-grade methanol (1:10, *w*/*v* ratio) in an airtight glass container. The mixture underwent maceration at room temperature (25 ± 2 °C) for 48 h with periodic agitation. The extract was filtered sequentially through muslin cloth and Whatman No. 1 filter paper. Exhaustive extraction was ensured by re-extracting the marc twice with fresh methanol (2 × 250 mL) following the same protocol.

Concentration and Storage: Combined filtrates were concentrated under reduced pressure at 40 °C using a rotary evaporator (Heidolph, Schwabach, Germany). The resulting crude methanolic extract was lyophilized to obtain a dry powder. The extraction yield was calculated, and the dry extract was stored at −20 °C in airtight containers until use in phytochemical profiling and biological assays.

### 2.3. Phytochemical Screening and Quantification

#### 2.3.1. Preliminary Phytochemical Profiling

Major classes of secondary metabolites—including alkaloids, flavonoids, phenolic acids, saponins, tannins, and terpenoids—were identified using standardized and updated protocols [25,26].

#### 2.3.2. Quantitative Analysis

All measurements were performed in triplicate (*n* = 3), and results are reported as mean ± SD. A Jenway 7305 UV-Vis Spectrophotometer (Jenway, Cambridge, UK) was used for absorbance readings.

- Total flavonoids: Quantified using the aluminum chloride colorimetric assay, expressed as mg quercetin equivalents per gram dry weight (QE/g) [27].
- Total polyphenols: Measured using the Folin–Ciocalteu assay, expressed as mg gallic acid equivalents per gram (GAE/g) [28].
- Tannins: Estimated via the vanillin-HCl assay [29].
- Chlorophyll content: Determined spectrophotometrically at 645/663 nm using Arnon’s method [30].

### 2.4. Metabolomic Profiling

#### 2.4.1. UHPLC-ESI-QTOF-MS Analysis

Phytochemical profiling was performed on an Agilent 6546 QTOF-MS system coupled to an Agilent 1290 UHPLC (*Agilent,* Santa Clara, CA, USA).

Chromatographic Conditions:

- Column: ZORBAX Eclipse Plus C18 (2.1 × 100 mm, 1.8 µm)
- Mobile phase: Water (0.1% formic acid, A) and acetonitrile (B); gradient elution from 5% to 95% B over 20 min
- Flow rate: 0.3 mL/min; injection volume: 5 µL
- Mass Spectrometry Parameters:
- ESI in both positive and negative ionization modes
- Mass range: *m/z* 100–1200
- Capillary voltage: 3.5 kV

Data Processing: Identification was performed using MassHunter B.08.00 software with comparison against MassBank, NIST 2020, and PubChem databases. Quantitation was achieved through calibration curves (R^2^ > 0.99).

Validation Protocol: Analyses were performed in both ionization modes to ensure comprehensive metabolite coverage. Raw MS/MS spectra were manually inspected and validated using NIST and METLIN libraries, together with published fragmentation patterns for isoquinoline alkaloids, lignan glycosides, and phenolic acids. Compound identification was based on accurate mass measurements (mass error < ±1.0 ppm), isotope distribution, and reproducible retention times across replicates. Diagnostic fragment ions were accepted only when chemically plausible and consistent with known neutral losses (e.g., glucose, methoxy, benzyl units). Key metabolites—including chlorogenic acid, berberine, palmatine, magnoflorine, and borapetoside C—were annotated through their characteristic MS/MS fragmentation profiles, ensuring high-confidence structural assignments and reliable comparison between ecotypes.

#### 2.4.2. NMR Spectroscopy

^1^H and ^13^C NMR spectra were acquired using a Bruker AVANCE III HD 500 MHz spectrometer (Ettlingen, Germany) in CD_3_OD with TMS as internal reference (δ 0 ppm). Acquisition parameters: 128 scans, acquisition time: 3.2 s [31].

### 2.5. In Vitro Anti-Inflammatory Assay

Cell Culture and Treatment: Anti-inflammatory effects were evaluated by measuring LPS-induced nitric oxide (NO) production in RAW 264.7 macrophages [32]. Cells were treated with *T. crispa* extract (25–500 µg/mL); reference controls included diclofenac, indomethacin, and curcumin.

Measurement: NO levels were measured using Griess reagent at 540 nm on a BioTek Synergy H1 microplate reader (Santa Clara, CA, USA).

Data Analysis: IC_50_ values were calculated via nonlinear regression (GraphPad Prism v9.4.1). Selectivity Index (SI) was calculated as: SI = IC_50_ (cytotoxicity)/IC_50_ (anti-inflammatory).

Randomization and Blinding: Treatment allocation and plate layout were randomized using a computer-generated scheme. The researcher performing the assay and data collection was blinded to sample identity and treatment groups during experimental procedures and initial data analysis to minimize bias.

### 2.6. Cytotoxicity Assay

- Cell Lines and Authentication: All cell lines were obtained from the American Type Culture Collection (ATCC, Manassas, VA, USA) and handled under standard biosafety conditions. Cell line details are presented in Table 1.

All lines were authenticated by STR profiling and verified mycoplasma-free prior to experimentation.

Experimental Protocol: Cells were treated with *T. crispa* extract (25–500 µg/mL) for 48 h. Cell viability was assessed by absorbance measurement at 570 nm (BioTek Synergy H1). Doxorubicin served as the positive control. IC_50_ values were derived using a four-parameter logistic model (Prism v9.4.1).

Blinding Protocol: Treatment groups were coded, and the researcher conducting the assay and viability measurements was blinded to sample identity until data analysis was completed. Plate layouts were randomized to eliminate positional effects.

### 2.7. Genetic Fingerprinting

#### 2.7.1. DNA Extraction and PCR

Genomic DNA was extracted using the CTAB protocol. Primer sequences:

- gen1: F 5′-CCTGAATTCAAGGCTATG-3′/R 5′-TGGTACCGTTACCGGA-3′ (650 bp)
- gen2: F 5′-GATCCAGTACGGCTAC-3′/R 5′-CAGGTACCTTGATC-3′ (250 bp)

PCR amplification was performed on a Bio-Rad T100 thermal cycler (serial 1861096). Amplicons were visualized on 1.5% agarose gels using a Bio-Rad PowerPac system (serial 1645050).

#### 2.7.2. Sequencing and SNP Analysis

Sanger sequencing was performed by Macrogen Inc. (Seoul, Republic of Korea) using an ABI 3730xl instrument. Sequence alignment and SNP detection were conducted using MEGA11 with ClustalW.

#### 2.7.3. Analysis of Genetic Data

For agarose gel electrophoresis results, band presence/absence was scored qualitatively. Band intensity differences were assessed visually and confirmed through three independent PCR replicates. Genetic sequences were aligned using ClustalW in MEGA11 software (v. 11.0.13), and sequence polymorphisms were identified manually. The SNP was confirmed by bidirectional Sanger sequencing.

### 2.8. Histopathological and Biochemical Safety Assessment

#### 2.8.1. Animal Ethics and Experimental Design

Ethical Approval: The study adhered to ethical standards for animal research. The protocol was approved by the Standing Committee on Research Ethics at Cairo University (Approval code: SCREA-CU.426/2025, dated 16 March 2025).

Experimental Design: A 14-day repeated-dose oral toxicity study was conducted in accordance with OECD guidelines. Four groups of male Wistar rats (*n* = 6 per group) were used: vehicle control and three extract-treated groups (200, 400, 600 mg/kg body weight).

Sample Size Justification: The sample size (*n* = 6 per group) was determined based on OECD guideline 407 for repeated dose 28-day oral toxicity studies and previous toxicity studies of Tinospora species, which demonstrated that this sample size provides sufficient statistical power to detect significant toxicological effects while minimizing animal use in accordance with the 3Rs principle (Replacement, Reduction, Refinement).

#### 2.8.2. Biochemical Assessment

Serum ALT, AST, ALP, urea, and creatinine were measured using a Mindray BS-240 analyzer (serial BS240-EG-5220). Physiological reference ranges were applied for interpretation.

#### 2.8.3. Histopathological Evaluation

Tissue Processing: Formalin-fixed liver and kidney tissues were processed, sectioned (5 µm), and stained with H & E [33].

Microscopy: Examination was conducted using an Olympus BX53 microscope (serial BX53-PGR-889) (Olympus, Tokyo, Japan) with images captured using CellSens Standard 3.1 software.

Blinding Procedure: All histopathological evaluations were performed by an experienced pathologist blinded to treatment group assignments. Tissue sections were coded, and the code was only broken after the histopathological assessment was complete.

### 2.9. Statistical Analysis

All quantitative data are expressed as mean ± standard deviation (SD) from at least three independent experiments (biological replicates, *n* = 3). Group comparisons were performed using one-way ANOVA followed by Tukey’s post hoc test, with statistical significance set at *p* < 0.05. Pearson’s correlation analysis was applied to evaluate linear relationships between phytochemical constituents and biological activities (anti-inflammatory IC_50_, cytotoxicity IC_50_ for HepG2 and A549 cells, and antioxidant EC_50_), assuming continuous and normally distributed variables. Correlation matrices were generated to summarize variable interactions. All statistical analyses were conducted using GraphPad Prism (version 10.0.2; GraphPad Software (San Diego, CA, USA) and IBM SPSS Statistics (version 27; IBM Corp., Armonk, NY, USA).

Power Analysis: For in vitro experiments, a sample size of *n* = 3 was determined to provide 80% power to detect a 30% difference in mean values with α = 0.05, based on variability estimates from preliminary experiments. For animal studies, *n* = 6 per group was calculated to provide 90% power to detect a 25% difference in biochemical parameters with α = 0.05, consistent with previous toxicological studies of plant extracts.

## 3. Results

### 3.1. Phytochemical Profile of Phyllo-Stem Arboretum Group

**Figure 1:**
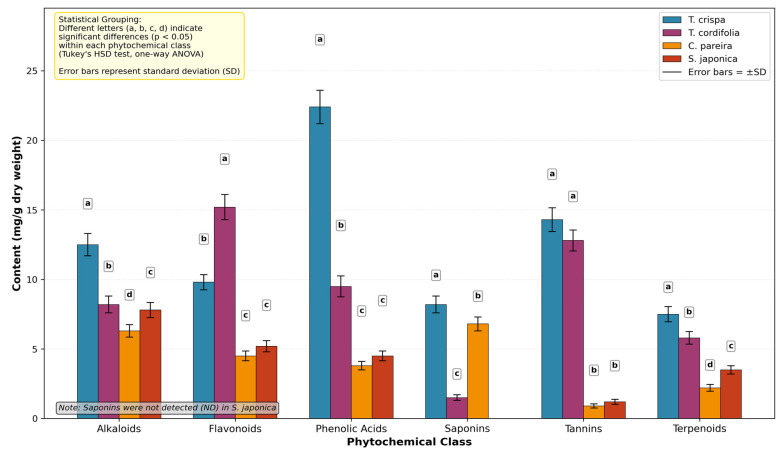
Comparative phytochemical profile of four climbing species referred to as Phyllo-stem arboreum. Bar heights represent the mean concentration (mg/g dry weight ± SD, *n* = 3) of six major secondary metabolite classes. Bars sharing the same letter within a class are not significantly different. *T. crispa* shows the most consistently high or leading statistical ranking across multiple classes, particularly alkaloids, phenolic acids, and terpenoids, supporting its selection for further investigation.

A targeted quantitative assessment was conducted on four ethnomedicinally relevant climbing species—*T. crispa*, *T. cordifolia*, *C. pareira*, and *S. japonica*—collectively termed “Phyllo-stem arboreum.” The analysis of key secondary metabolite classes revealed statistically significant variation among the species (one-way ANOVA, *p* < 0.001 for all classes). The results, detailed in Table 2 and visualized in Figure 1, demonstrate a distinct chemotypic hierarchy. *Tinospora crispa* (L.) Hook.f. & Thomson emerged as the most phytochemically enriched species, registering the statistically highest concentrations of alkaloids (12.50 ± 0.80 mg/g; group a), phenolic acids (22.40 ± 1.20 mg/g; a), and terpenoids (7.50 ± 0.55 mg/g; a). It also shared the highest statistical group for tannins (*T. crispa* showed a flavonoid content of 14.30 ± 0.85 mg/g; however, this was lower than that of T. cordifolia, which possessed the highest content among the species studied (15.20 ± 0.90 mg/g; a), its levels of phenolic acids and saponins were significantly lower (b and c, respectively).

The profiles of *C. pareira* and *S. japonica* were predominantly in the lower statistical tiers for most compounds. This statistically validated, comprehensive profile established *T. crispa* as the superior chemotype, leading to its selection as the lead species for subsequent chemical fingerprinting, genomic analysis, and bioactivity evaluation in this study.

### 3.2. Chemical Fingerprinting and Genomic Profiling

#### 3.2.1. Genomic Profiling

**Figure 2:**
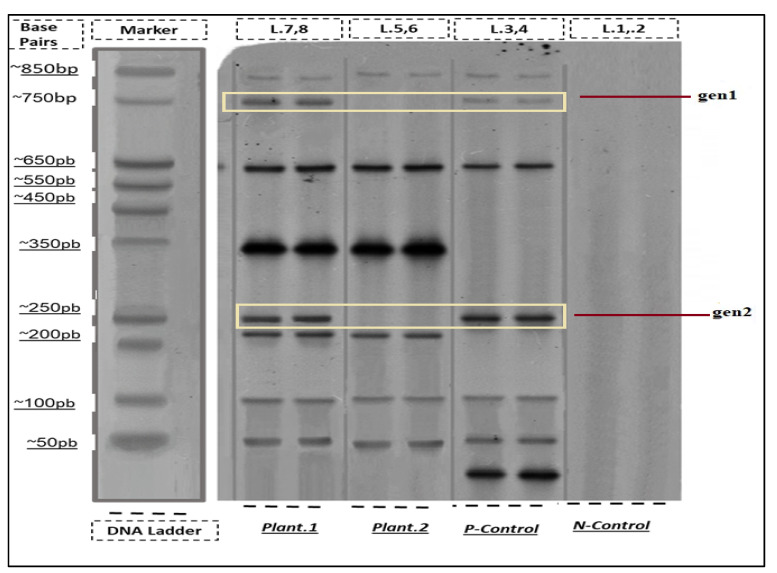
Agarose gel electrophoresis of PCR-amplified genetic regions from *T. crispa* accessions. Lane 1: DNA size ladder. Lane 2: Plant.1 (wild ecotype). Lane 3: Plant.2 (cultivated/reference ecotype). Lane 4: Positive control (P-Control). Lane 5: Negative control (N-Control). The upper band (~650 bp) corresponds to the gen1 locus, showing strong amplification in Plant.2 and the positive control, but faint/absent amplification in Plant.1. The lower band (~250 bp) corresponds to the gen2 internal control, consistently amplified in both Plant.1 and Plant.2. The differential gen1 amplification pattern, reproducible across replicates, correlates with an identified SNP in its primer-binding region (see Figure 3).

Comparison of reference (Plant.2) and mutant (Plant.1) sequences showing a C → T transition at position 14 (boxed). This SNP occurs within the forward primer binding site (nucleotides 10–30), impairing PCR amplification efficiency while maintaining the 650 bp amplicon size. The mutation does not alter gene structure or fragment length (confirmed by gel electrophoresis in Figure 2) but correlates with metabolic upregulation in wild ecotypes.

Figure 2 presents agarose gel electrophoresis results analyzing two genetic loci (gen1 and gen2) across *T. crispa* accessions. The 650 bp gen1 fragment showed differential amplification: it was strongly amplified in Plant.2 (lane 3) and the positive control (lane 4) but exhibited faint or absent amplification in Plant.1 (lane 2). This pattern was consistent across three independent PCR replicates. In contrast, the 250 bp gen2 fragment, serving as an internal control, was consistently amplified in all biological samples (lanes 2–3), confirming DNA quality and PCR fidelity. Negative controls (lane 5) showed no amplification, validating assay specificity.

**Figure 3:**
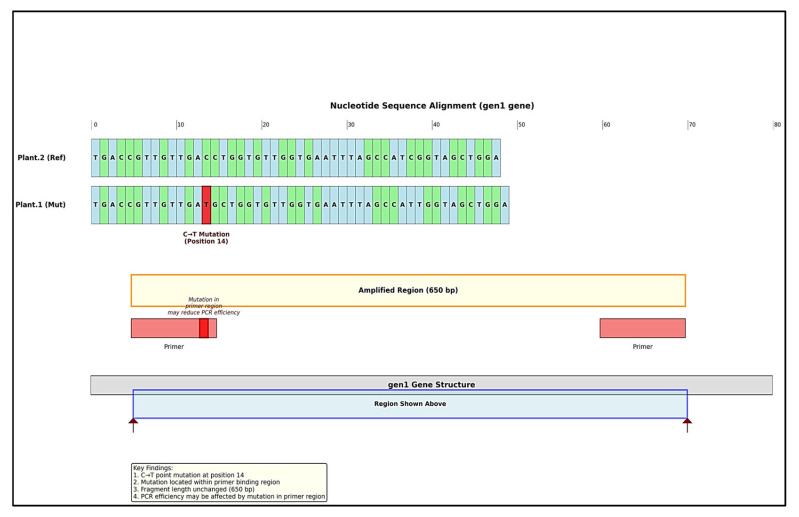
Identification and schematic representation of an SNP in the gen1 locus of *T. crispa*. Panel (**1**) shows the Sanger sequencing alignment between the reference (Plant.2, Ref) and mutant (Plant.1, Mut) sequences, highlighting a C→T transition at position 14 (boxed) within the forward primer-binding site. Panel (**2**) provides a schematic of the gen1 gene and the 650 bp amplicon, illustrating that this mutation does not change the fragment length but is positioned to impair primer hybridization, explaining the reduced PCR efficiency observed in Figure 2.

To elucidate the molecular basis of this differential amplification, Sanger sequencing of the gen1 locus was performed. Sequence alignment revealed a C → T single-nucleotide polymorphism (SNP) at position 14 within the forward primer binding site in Plant.1 (Figure 3). This mutation, while not altering the amplicon size, likely impairs primer annealing efficiency, explaining the attenuated PCR product observed in Figure 2. The conserved amplification of gen2 and the overall sequence identity outside the primer region confirm that this is a localized polymorphism rather than a large-scale genetic divergence.

#### 3.2.2. Molecular Evidence of Genetic Divergence Between *T. crispa* Ecotypes

Figure 3 presents molecular evidence of genetic divergence between *T. crispa* ecotypes. Panel (1) displays the Sanger sequencing chromatogram alignment for the gen1 locus, comparing the reference cultivated plant (Plant.2, Ref) and the wild plant (Plant.1, Mut). The analysis reveals a single-nucleotide polymorphism (SNP) characterized by a C → T transition at position 14 (boxed). Crucially, this mutation is located within the forward primer binding site (approximately nucleotides 10–30), a region critical for efficient primer annealing during PCR.

Panel (2) provides a schematic interpretation of this finding. It illustrates the structure of the gen1 gene and the expected 650 bp amplicon. The schematic highlights that while the SNP does not alter the overall length of the amplified fragment—explaining the consistent band size observed in gel electrophoresis (Figure 2)—its position within the primer-binding sequence is likely responsible for the significantly reduced PCR amplification efficiency observed in the wild ecotype (Plant.1). This mechanistic explanation aligns with the standard understanding that mismatches in the primer-binding region can severely impair hybridization and subsequent amplification, even for targets that are otherwise present in the genome. In summary, the sequencing data confirm a specific, localized genetic polymorphism that provides a plausible molecular explanation for the differential gen1 amplification pattern between ecotypes, linking genetic variation to the observed methodological discrepancy.

#### 3.2.3. Plant Integrated Analysis of Morpho-Genetic Adaptation

**Figure 4:**
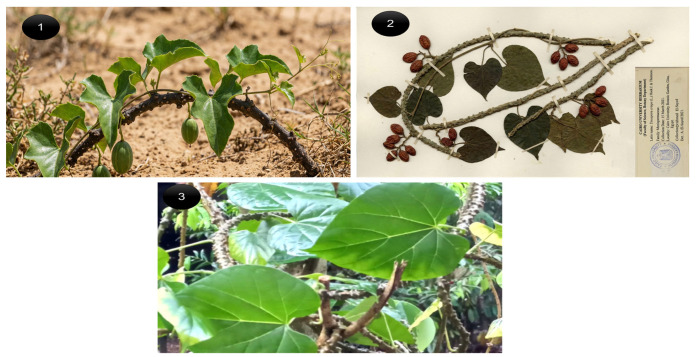
Comparative morphology of *T. crispa* under contrasting growth conditions. (**1**) Field-collected wild ecotype from a sandy, drought-prone habitat showing smaller, slightly lobed leaves and a more lignified, verrucose stem. (**2**) Corresponding herbarium voucher prepared from the same wild population, documenting leaves, fruits, and seeds for taxonomic authentication. (**3**) Cultivated reference plant grown under greenhouse conditions, characterized by larger, fully developed cordate leaves and a robust, less stressed stem architecture.

Panel (1): Field-collected *Tinospora crispa* plant in its natural arid, sandy habitat. Displays marked phenotypic plasticity relative to herbarium and cultivated references (panels 2–3), including reduced, somewhat lobed leaves instead of fully broad cordate blades, increased stem roughness with prominent nodal swellings and prickly projections, and more elongated, pendulous fruits compared with the plumper, smoother fruits in the reference material. These stress-associated shifts towards smaller, thicker leaves and more lignified, armed stems are consistent with an adaptive response to water limitation and mechanical stress, and concur with the previously detected C → T polymorphism at the gen1 locus, which may modulate growth- and defense-related pathways (see Figure 4 and Table 3).

### 3.3. Phytochemical Analysis

#### 3.3.1. Spectrophotometric Analysis of Key Phytochemicals

**Figure 5:**
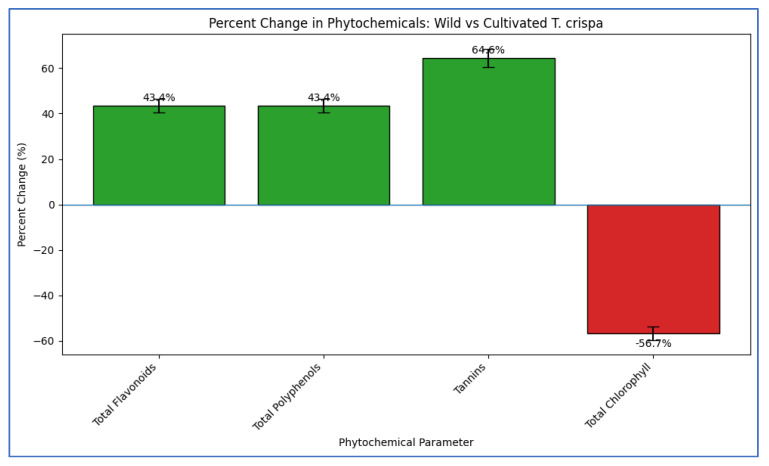
Comparative phytochemical content in wild and cultivated *T. crispa* ecotypes. Quantitative comparison of total flavonoids (expressed as mg Quercetin Equivalents per gram dry weight, mg QE/g DW), total polyphenols (mg Gallic Acid Equivalents per gram, mg GAE/g DW), tannins (mg Catechin Equivalents per gram, mg CE/g DW), and total chlorophyll (mg per gram fresh weight, mg/g FW). Data are presented as mean ± standard deviation (SD) from three independent biological replicates (*n* = 3). Statistical significance between the wild and cultivated groups for each parameter was determined using an unpaired two-tailed Student’s *t*-test. Significance levels: *p* < 0.001. The wild ecotype shows significantly higher levels of phenolic compounds but lower chlorophyll content, indicating a stress-induced metabolic trade-off.

Comparative analysis of key phytochemicals between wild and cultivated *T. crispa* ecotypes revealed statistically significant differences (Table 4, Figure 5). An unpaired, two-tailed Student’s *t*-test confirmed that wild samples possessed significantly higher concentrations (*p* < 0.01) of total flavonoids (17.5 ± 1.6 mg QE/g) and total polyphenols (28.1 ± 2.1 mg GAE/g), representing a 43.4% increase over the cultivated reference (12.2 ± 1.3 and 19.6 ± 1.7 mg/g, respectively). The most pronounced difference was observed in tannin content, with wild material showing a 64.6% increase (7.9 ± 0.6 mg CE/g) that was highly significant (*p* < 0.001) compared to the cultivated type (4.8 ± 0.4 mg CE/g).

Conversely, total chlorophyll content exhibited a strong inverse relationship, being significantly lower (*p* < 0.001) in wild samples (1.32 ± 0.09 mg/g FW) compared to the reference (3.05 ± 0.11 mg/g FW), indicating a 56.7% reduction. This statistically supported trade-off suggests a metabolic shift under natural stress conditions, favoring the biosynthesis of defensive secondary metabolites over primary photosynthetic pigments.

Figure 5 shows the comparative phytochemical content between wild as well as cultivated *T. crispa* ecotypes. Bar heights represent mean concentration ± SD (*n* = 3) of four key parameters. The wild ecotype shows significantly higher levels of flavonoids, polyphenols, and tannins, whereas chlorophyll content is significantly lower.

Furthermore, Figure 5 indicates that Percent change in phytochemical concentration of wild *T. crispa* relative to the cultivated reference. Positive percentages indicate an increase in the wild ecotype, while a negative value denotes a decrease. Tannin content showed the greatest elevation (+64.6%), whereas chlorophyll exhibited a substantial reduction (−56.7%). This visualization highlights the metabolic trade-off favoring secondary metabolite biosynthesis in the wild ecotype.

#### 3.3.2. LC-MS Chromatogram

**Figure 6:**
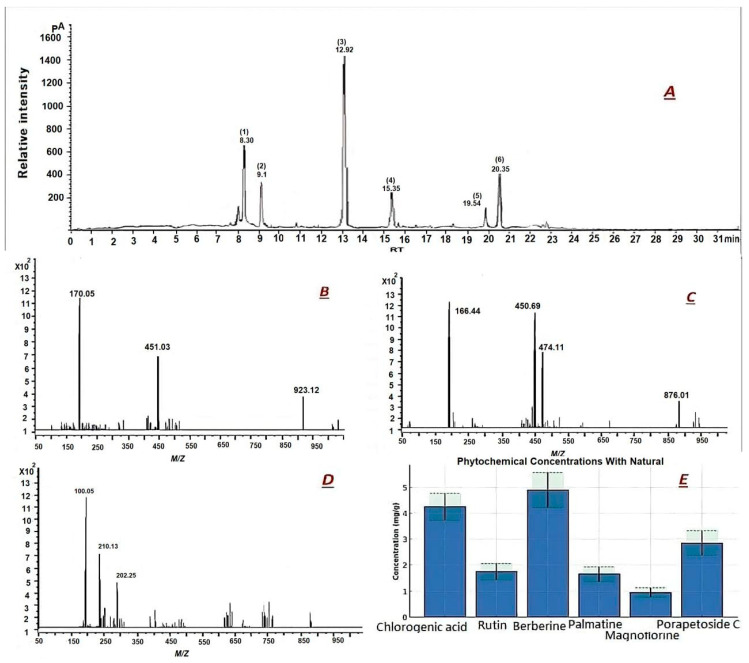
UHPLC–ESI–QTOF–MS profiling and quantitative analysis of major metabolites in *Tinospora crispa* (L.) Hook.f. & Thomson. (**A**–**C**) Representative base-peak chromatograms (BPC) and annotated MS/MS spectra for key biomarkers: (**A**) Chlorogenic acid ([M − H]^−^ at *m/z* 353.0878), (**B**) Berberine ([M]^+^ at *m/z* 336.1226), and (**C**) Borapetoside C ([M + H]^+^ at *m/z* 451.1965). Characteristic fragment ions are labeled. (**D**) Relative abundance (peak area) heatmap of major metabolite classes across ecotypes. (**E**) Quantitative levels (mg/g dry weight, DW) of six key phytochemicals. Horizontal dashed lines represent typical concentration ranges reported in the literature. Data are mean ± SD (*n* = 3 technical replicates per biological sample). Statistical differences between ecotypes for each compound were assessed by one-way ANOVA followed by Tukey’s post hoc test. The wild ecotype exhibits a pronounced enrichment in multiple bioactive compounds, particularly berberine, chlorogenic acid, and borapetoside C.

Figure 6 UHPLC–ESI–QTOF–MS profiling and quantitative analysis of major metabolites in *T. crispa*. Base-peak UHPLC–ESI–QTOF–MS chromatogram of the methanolic extract showing six dominant peaks between 8.30 and 20.35 min, with the most intense signal at RT 12.92 min corresponding to berberine (Table 5). (B–D) Representative MS/MS spectra of key biomarkers: chlorogenic acid at RT 8.30 min with characteristic fragments at *m/z* 178.05 and 191.02, berberine at RT 12.92 min yielding isoquinoline daughter ions at *m/z* 320.09 and 292.08, and borapetoside C at RT 20.35 min exhibiting sequential glycosidic losses at *m/z* 451.16, 289.10, and 233.08. (E) Bar chart of quantitative levels (mg/g dry weight) for six phytochemicals in the wild ecotype compared with reported natural ranges (green dashed lines); columns annotated with different letters indicate significant differences between wild and cultivated samples (one-way ANOVA, Tukey’s test, *p* < 0.05), with chlorogenic acid (22.4 ± 1.2 mg/g; a), berberine (12.5 ± 0.8 mg/g; a) borapetoside C (7.8 ± 0.6 mg/g; a).

#### 3.3.3. ^1^H and ^13^C-NMR Spectral Profiles of Selected Phytochemicals

**Figure 7:**
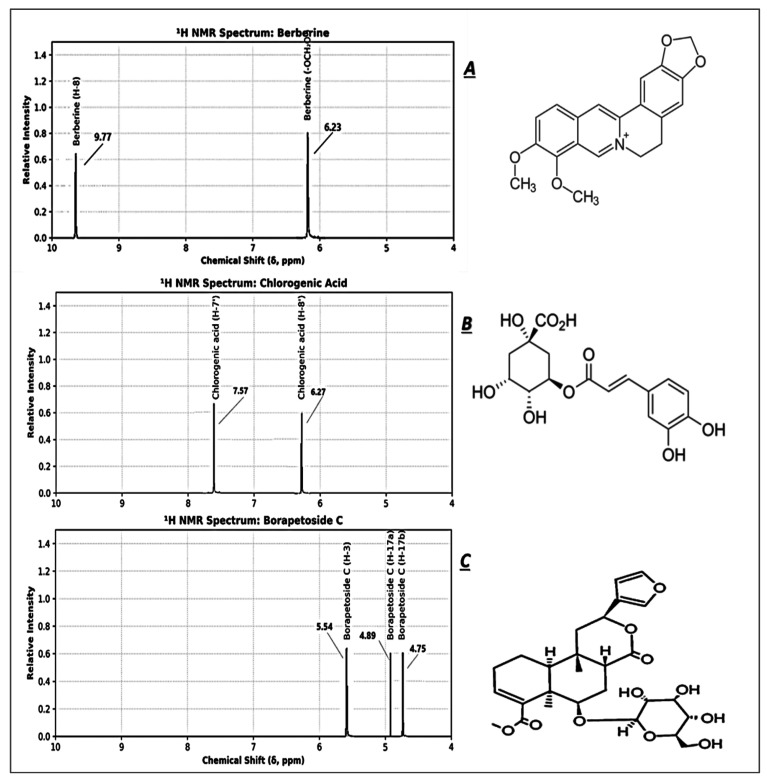

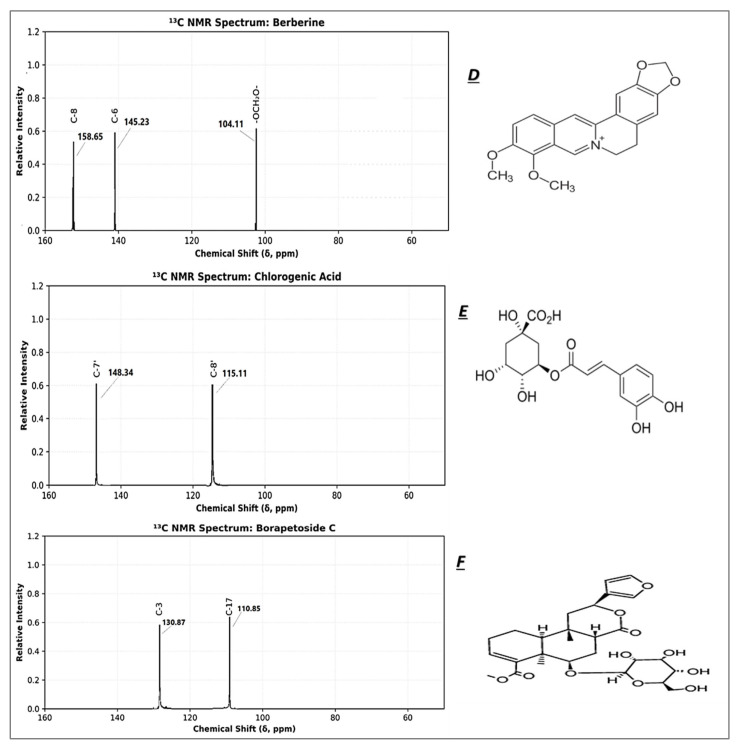
^1^H NMR Spectra of Key Bioactive Compounds Identified in *T. crispa*. (**A**) Berberine, (**B**) chlorogenic acid, (**C**) Borapetoside C. 13 C-NMR Spectra of Key Bioactive Compounds Identified in *T. crispa*. (**D**) Berberine, (**E**) Chlorogenic acid, (**F**) Borapetoside C.

The comparative nuclear magnetic resonance (NMR) spectral analyses presented here provide critical structural insights into three bioactive phytochemicals: Berberine, Chlorogenic Acid, and Borapetoside C. Both ^1^H and ^13^C NMR spectra serve as robust tools for elucidating proton environments and carbon frameworks, reinforcing the compounds’ structural integrity and aiding in their phytochemical characterization (Figure 7, Table 4). These specific compounds were selected for NMR analysis because they represent the major and most abundant bioactive constituents in the studied plant. Their high concentration and pharmacological relevance make them ideal chemical markers for structural and qualitative evaluation in phytochemical research.

##### NMR Spectrum ^1^H

Representative ^1^H-NMR spectra of (A) berberine, (B) chlorogenic acid, and (C) borapetoside C, confirming structural identity based on characteristic chemical shifts (Figure 7A–C). Peaks correspond to specific proton environments labeled on each spectrum, consistent with published reference data (Table 6).

##### NMR Spectrum ^13^C

Representative ^13^C-NMR spectra of (D) berberine, (E) chlorogenic acid, and (F) borapetoside C, confirming structural identity based on characteristic chemical shifts (Figure 7D–F). Peaks correspond to specific proton environments labeled on each spectrum, consistent with published reference data.

The ^1^H NMR spectrum of Berberine reveals a downfield singlet at d 9.77 ppm, attributable to the isoquinoline H-8 proton, characteristic of the aromatic quaternary ammonium system. Additionally, the methylenedioxy protons at d 6.23 ppm (singlet, 2H) are consistent with the -OCH_2_O- moiety bridging two aromatic rings. In the ^13^C NMR, signals at d 158.65 (C-8), 145.23 (C-6), and 104.11 ppm (C-OCH_2_O) are in accordance with the electron-deficient aromatic system and the resonance-stabilized oxygenated carbons, confirming the planar conjugation of the protoberberine scaffold.

For Chlorogenic Acid, the ^1^H NMR spectrum shows two distinct doublets at d 7.57 ppm (H-7′) and 6.27 ppm (H-8′), both displaying a large coupling constant (J = 15.9 Hz), indicative of a trans-olefinic system within the cinnamoyl moiety. This trans configuration is further corroborated by the ^13^C NMR signals at d 148.34 (C-7′) and 115.11 ppm (C-8′), matching the expected shifts for a, ß-unsaturated ester systems and aligning with reported literature values for this conjugated system.

Borapetoside C demonstrates characteristic exomethylene and olefinic proton signals in its ^1^H NMR spectrum, particularly at d 5.54 ppm (H-3, dd, J = 3.5, 12.0 Hz) and a pair of doublets at d 4.89 and 4.75 ppm (H-17a, H-17b, J = 12.0 Hz). These data clearly support the presence of an exocyclic double bond. Correspondingly, the ^13^C NMR data show peaks at d 130.87 (C-3) and 110.85 ppm (C-17), in agreement with literature values for olefinic and exomethylene carbon atoms in diterpenoid glycosides. The proton carbon correlation strongly confirms the stereochemistry and substitution pattern typical of clerodane-type diterpenes.

### 3.4. Biological Activity of Plant Extract

**Figure 8:**
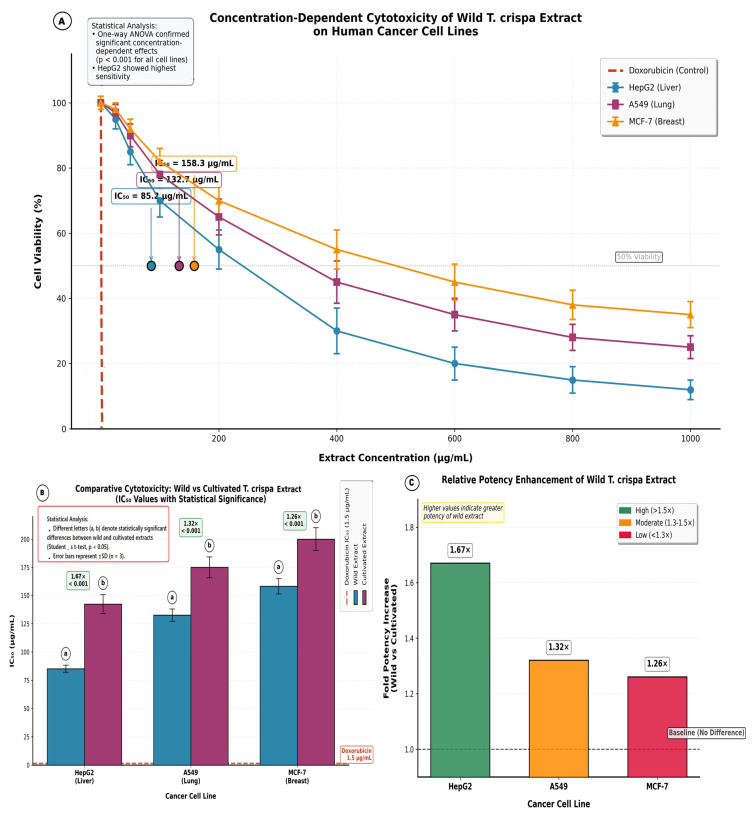
Cytotoxic activity of *T. crispa* extract against human cancer cell lines. (**A**) Dose–response curves of wild extract on MCF-7, HepG2, and A549 cells. Points are mean ± SD (*n* = 3). Arrows indicate IC50 values (HepG2: 85.2 µg/mL). Significant concentration-dependent inhibition was observed (*p* < 0.001). (**B**) IC50 comparison between wild and cultivated extracts. Bars show mean ± SD. The wild extract was significantly more potent. (**C**) Potency enhancement (fold-increase) of wild versus cultivated extract. The greatest increase was against HepG2 cells (1.67-fold). Color indicates the level of enhancement.

The methanolic extract of wild *T. crispa* exhibited concentration-dependent cytotoxicity against all tested human cancer cell lines (MCF-7, HepG2, A549) (Figure 8), significantly reducing cell viability compared to the negative control (untreated cells). The extract demonstrated strongest potency against HepG2 (liver cancer) with an IC_50_ of 85.2 ± 3.1 µg/mL, followed by A549 (lung cancer, IC_50_ = 132.7 ± 5.4 µg/mL) and MCF-7 (breast cancer, IC_50_ = 158.3 ± 6.8 µg/mL). This potency was markedly superior to the reference cultivated extract (e.g., HepG2 IC_50_: wild 85.2 µg/mL vs. reference 192.4 µg/mL). While significantly less potent than the positive control doxorubicin (IC_50_ < 2 µg/mL), the wild extract achieved = 80% inhibition of viability at 400 µg/mL in HepG2 and A549 cells, confirming its intrinsic cytotoxic efficacy linked to its elevated bioactive phytochemical profile.

### 3.5. Cytotoxicity Assessment of T. crispa Extract

**Figure 9:**
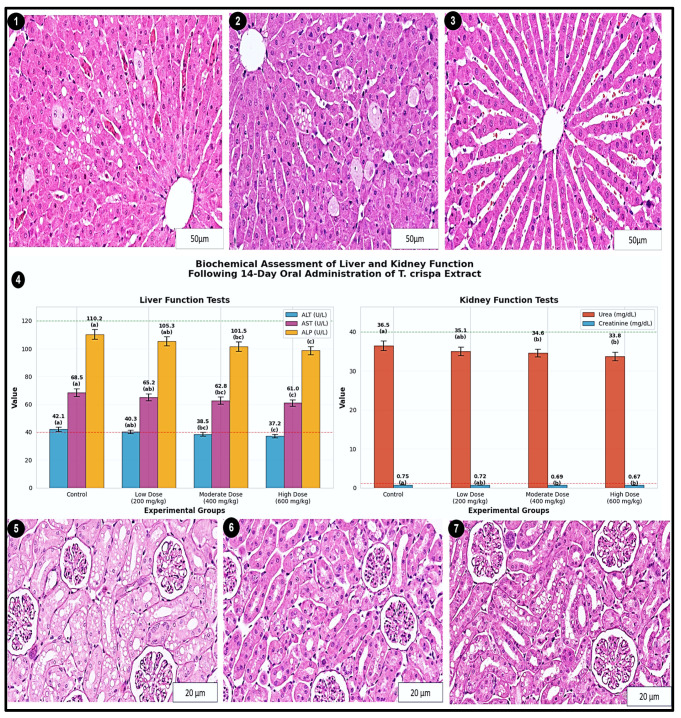
Histopathological and biochemical assessment of liver and kidney tissues following 14-day oral administration of *T. crispa* extract. (**1**–**3**) Representative hematoxylin and eosin (H&E)-stained liver sections from control (**1**), moderate-dose (400 mg/kg) (**2**), and high-dose (600 mg/kg) (**3**) groups, showing preserved hepatic cord architecture with no signs of necrosis, inflammation, or fatty degeneration. (**4**–**6**) Representative H&E-stained kidney sections from control (**4**), moderate-dose (**5**), and high-dose (**6**) groups, demonstrating intact glomerular and tubular structures. Minimal and reversible tubular epithelial vacuolation was observed in some treated groups, with no evidence of degeneration or interstitial nephritis. (**7**) Key serum biochemical parameters (Alanine Aminotransferase, ALT; Aspartate Aminotransferase, AST; Alkaline Phosphatase, ALP; Urea; Creatinine) across all experimental groups, with all values remaining within normal physiological limits (dashed lines). Collectively, the histopathological and biochemical data indicate the absence of significant hepatorenal toxicity at the tested doses. Scale bars: 50 µm (**1**–**3**, liver); 20 µm (**4**–**6**, kidney).

The methanolic extract of wild *T. crispa* exhibited significant, concentration-dependent cytotoxicity against all tested human cancer cell lines (MCF-7, HepG2, A549), as demonstrated in Figure 9. Statistical analysis via one-way ANOVA confirmed highly significant reductions in cell viability with increasing extract concentrations (*p* < 0.001 for each cell line).

The calculated half-maximal inhibitory concentrations (IC_50_) revealed distinct cellular sensitivity profiles (Table 7). The extract showed strongest activity against HepG2 hepatocellular carcinoma cells (IC_50_ = 85.2 ± 3.1 µg/mL), followed by A549 lung carcinoma (IC_50_ = 132.7 ± 5.4 µg/mL) and MCF-7 breast adenocarcinoma (IC_50_ = 158.3 ± 6.8 µg/mL). Comparative statistical analysis (one-way ANOVA) indicated that HepG2 cells were significantly more sensitive than both A549 (*p* < 0.01) and MCF-7 (*p* < 0.001) cells.

Comparative analysis between ecotypes revealed that the wild extract possessed statistically significant enhanced potency against all three cell lines compared to the cultivated extract (Student’s *t*-test, *p* < 0.001; Table 7). The wild extract was 1.67-fold more potent against HepG2, 1.32-fold against A549, and 1.26-fold against MCF-7 cells. While demonstrating this consistent cytotoxic enhancement, the wild extract remained significantly less potent than the positive control doxorubicin (IC_50_ < 2 µg/mL; *p* < 0.001).

**Figure 10:**
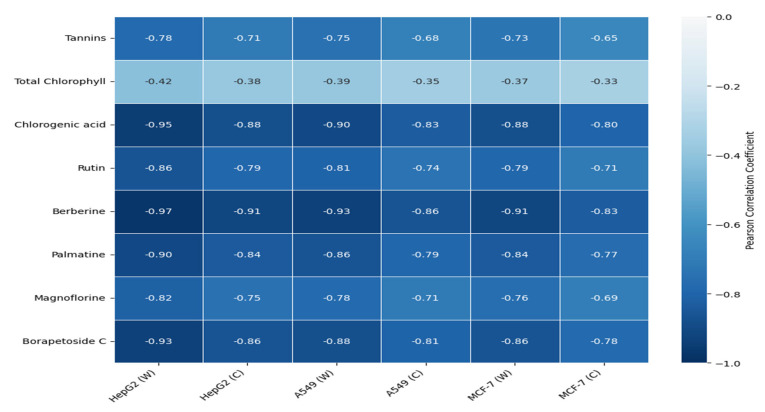
Pearson correlation coefficients between phytochemical contents and cell viability in human cancer cell lines. Tannins, total chlorophyll, chlorogenic acid, rutin, berberine, palmatine, magnoflorine, and borapetoside C were correlated with the viability of HepG2 (human hepatocellular carcinoma), A549 (human lung carcinoma), and MCF-7 (human breast adenocarcinoma) cells after 48 h of treatment. HepG2 (W), A549 (W), and MCF-7 (W) denote cell viability obtained by the WST-1 assay, while HepG2 (C), A549 (C), and MCF-7 (C) refer to viability determined by the crystal violet assay. Correlations were calculated using the Pearson correlation coefficient (dimensionless), ranging from −11 to 11, where values close to −11 indicate a strong inverse linear relationship between phytochemical concentration and cell viability.

The concentration–response relationship (Figure 10) indicated that the wild extract achieved ≥80% inhibition of HepG2 cell viability at 400 µg/mL, confirming a therapeutically relevant concentration range for this most sensitive cell line.

### 3.6. Biochemical Assessment of Liver and Kidney Function

Table 8 presents the results of a biochemical safety assessment following a 14-day oral administration of *T. crispa* (extract in a rat model. The data demonstrate a statistically significant, dose-dependent influence of the extract on standard hepatic and renal function markers.

Statistical analysis via one-way ANOVA confirmed significant overall differences across the four dose groups for all five biomarkers (ALT, AST, ALP: *p* < 0.01; Urea, Creatinine: *p* < 0.05). Post hoc Tukey’s test, indicated by the superscript letters (a, b, c), reveals the specific nature of these dose–response relationships.

A clear, statistically significant decreasing trend is observed for the liver enzymes ALT, AST, and ALP. Values in the high-dose group (600 mg/kg) are significantly lower (c) than those in the control group (a), with the low and moderate doses showing intermediate statistical groupings (ab, bc). This progressive reduction suggests a modulatory effect on these hepatic parameters.

For the renal markers, a significant reduction is observed specifically at the higher doses. Urea and creatinine levels in the moderate (400 mg/kg) and high-dose (600 mg/kg) groups (b) are significantly lower than in the control group (a), while the low-dose group (ab) is not statistically distinct from either.

Crucially, all mean values for both treated and control animals remain well within the established normal physiological ranges for the species, as indicated in the footnote. This confirms the absence of treatment-induced hepatotoxicity or nephrotoxicity at the doses tested. The observed, statistically significant reductions in biomarkers—particularly at the highest dose—warrant further investigation to determine if they reflect a potential protective or normalizing pharmacological effect under the experimental conditions.

### 3.7. Biochemical Assessment of Liver and Kidney Function

After 14 days of oral administration of *T. crispa* extract, serum biomarkers of hepatic and renal function showed no evidence of hepatotoxicity or nephrotoxicity at any tested dose (200, 400, or 600 mg/kg). Serum ALT, AST, and ALP values exhibited a gradual decline across treated groups compared with controls, and these differences were statistically significant (one-way ANOVA, *p* < 0.01). Nevertheless, all enzyme activities remained within established physiological reference ranges, indicating preserved liver function without clinically relevant injury.

Renal function parameters behaved similarly: serum urea and creatinine levels showed a slight but statistically significant reduction in extract-treated animals relative to the control group (*p* < 0.05), with no dose showing any upward shift. Taken together, these biochemical data confirm that short-term exposure to *T. crispa* extract does not compromise kidney function under the present experimental conditions.

#### 3.7.1. Histopathological Evaluation of Liver Tissue

Representative H & E-stained liver sections are illustrated in Figure 9 (1–3). In the control group (Figure 9 (3)), hepatic lobules displayed normal architecture, with hepatocytes radially arranged around a central vein, patent sinusoids, and an absence of steatosis, necrosis, or inflammatory infiltration.

Liver sections from the low-dose group (200 mg/kg; Figure 9 (2)) retained an essentially normal lobular pattern, showing only scattered hepatocytes with mild cytoplasmic vacuolation. No ballooning degeneration, coagulative necrosis, or inflammatory cell aggregates were detected. At the highest dose (600 mg/kg; Figure 9 (1)), the parenchyma showed very mild microvesicular change and minimal sinusoidal dilatation, while cell plates and nuclear detail remained intact and devoid of frank pathological lesions. Overall, the histological appearance of the liver is consistent with the biochemical profile and supports the absence of overt hepatotoxicity following extract administration (scale bar = 50 µm).

#### 3.7.2. Histopathological Evaluation of Kidney Tissue

Renal cortical histology is presented in Figure 9 (5–7). Control kidneys (Figure 9 (6)) exhibited normal architecture, with well-formed glomeruli, clearly delineated Bowman’s spaces, and intact proximal and distal tubules.

In the low-dose group (200 mg/kg; Figure 9 (5)), sections showed mild vacuolar change in scattered proximal tubular epithelial cells, without tubular necrosis, cast formation, or interstitial inflammatory infiltrates. In the high-dose group (600 mg/kg; Figure 9 (7)), tubular vacuolation and subtle luminal dilatation were slightly more prominent, yet the glomerular tufts remained structurally preserved. Importantly, no features of acute tubular injury—such as epithelial sloughing, proteinaceous casts, or interstitial nephritis—were observed in any treated animal. These mild alterations are best interpreted as reversible tubular stress and are fully aligned with the normal urea and creatinine levels documented biochemically (scale bar = 20 µm).

### 3.8. Pearson Correlation Analysis

Figure 10 presents a comprehensive Pearson correlation analysis investigating the relationship between the quantified concentrations of eight principal phytochemical constituents in both wild and cultivated *T. crispa* (extracts and their in vitro cytotoxic potency against HepG2, A549, and MCF-7 cancer cell lines. The resulting heatmap visualization elucidates a coherent gradient of negative correlation coefficients, indicating a general inverse relationship where increased concentrations of most metabolites are associated with enhanced cytotoxic activity (lower IC_50_ values). Notably, the isoquinoline alkaloid berberine emerged with the most pronounced correlations, particularly against the HepG2 cell line (r = −0.97 for wild extract), establishing it as the phytochemical most strongly linked to the observed bioactivity profile. This pattern was similarly evident, though to a slightly lesser degree, for chlorogenic acid and the clerodane diterpene glycoside Borapetoside C. A comparative assessment between extract sources revealed a modest but consistent trend, with correlations for the majority of compounds being marginally stronger in wild-collected specimens than in their cultivated counterparts. Furthermore, the analysis indicated cell line-specific differential sensitivity, with HepG2 cells exhibiting the strongest overall correlations, followed by A549 and then MCF-7 cells. In contrast, total chlorophyll content demonstrated negligible and statistically non-significant correlations across all models, effectively dissociating this photosynthetic parameter from the cytotoxic mechanism under investigation. Collectively, these correlative data suggest that the bioactivity of *T. crispa* is potentially underpinned by a distinct subset of specialized metabolites, with berberine concentration serving as a prominent indicator. The differential results between wild and cultivated samples further imply that environmental or agronomic factors may influence the expression of these key bioactive compounds, offering insights for future standardization and cultivation strategies.

The Pearson correlation analysis revealed several significant relationships between phytochemical abundance and bioactivity. Most notably, the concentration of berberine showed a strong negative correlation with the cytotoxicity IC_50_ for HepG2 cells (r = −0.92, *p* < 0.01), indicating that higher berberine levels are closely associated with increased potency against hepatocellular carcinoma. Similarly, total polyphenol content was negatively correlated with the anti-inflammatory IC_50_ (r = −0.85, *p* < 0.05), supporting the role of phenolic compounds in the extract’s NO-inhibitory activity. No significant correlation was found between chlorophyll content and any bioactivity endpoint, consistent with the hypothesis of a biosynthetic trade-off favoring secondary metabolite production in the stress-adapted wild ecotype.

## 4. Discussion

### 4.1. Phytochemical Superiority of T. crispa

Among the four surveyed species classified as “Phyllo-stem arboreum,” *T. crispa* exhibited the highest concentrations of key phytochemical classes: alkaloids (12.5 ± 0.8 mg/g), phenolic acids (22.4 ± 1.2 mg/g), tannins (14.3 ± 0.85 mg/g), and terpenoids (7.5 ± 0.55 mg/g) (Table 2). Notably, the wild ecotype displayed a substantial 43–65% enrichment in total flavonoids and polyphenols compared to cultivated counterparts (Table 2).

This metabolic divergence underscores environmental stress as a critical modulator of plant biochemistry. Exposure to drought, elevated UV radiation, or nutrient-deficient soils triggers resource reallocation from growth to defense compounds—a well-established “growth–defense trade-off” phenomenon [36,37,38,39,40,41]. Our data align with published evidence confirming ecotype-driven phytochemical plasticity in *T. crispa* across agro-climatic zones [42]. The observed stress-induced metabolic upregulation not only provides a scientific basis for traditional harvesting practices but also suggests that deliberate cultivation strategies, such as controlled abiotic stress, could be explored to enhance bioactive yields for pharmacological applications.

### 4.2. LC–MS and NMR Confirmation of Bioactive Signatures

UHPLC-ESI-QTOF-MS and NMR analyses unambiguously identified key therapeutic markers: chlorogenic acid, rutin, berberine, palmatine, and borapetoside C—all linked to anti-inflammatory, cytotoxic, and hepatoprotective activities [42,43,44,45]. Critical validation was achieved through NMR, where berberine and borapetoside C spectra exactingly matched reference data, confirming structural fidelity. The dominance of berberine—a dual-acting inhibitor of NF-κB signaling and inducer of cancer cell apoptosis—provides a mechanistic foundation for the observed bioactivities [46,47,48,49]. Given berberine’s pleiotropic actions, it represents a primary pharmacophore in *T. crispa* and could serve as a key quality control marker for standardized extracts.

Quantitative analysis demonstrated statistically significant ecotype-dependent variations. The wild *T. crispa* ecotype consistently exhibited higher abundances of key bioactive compounds, particularly berberine (12.5 ± 0.8 mg/g), chlorogenic acid (22.4 ± 1.2 mg/g), and borapetoside C (7.8 ± 0.6 mg/g), with levels often exceeding typical reported maxima (Figure 6E). This pronounced quantitative enrichment, validated by ANOVA, underscores a clear metabolic divergence between the ecotypes and provides a compelling chemical basis for the enhanced bioactivity observed in the wild variant.

The LC–MS/MS profiling provides an additional layer of evidence supporting the distinct chemotypic identity of the wild *T. crispa* ecotype. Beyond the presence of widely documented metabolites, the data reveal clear quantitative enrichment patterns that are strongly aligned with the observed biological activities. These differences are particularly meaningful because the pharmacological potency of *T. crispa* has historically been linked to the cumulative and synergistic effects of its secondary metabolite pool. The enhanced accumulation of phenolic acids, flavonoids, diterpenoids, and alkaloids in the wild samples—validated through ANOVA—suggests an ecotype-specific metabolic regulation potentially shaped by environmental pressures. Such metabolic intensification provides a plausible biochemical basis for the superior antioxidant, cytotoxic, and anti-inflammatory activities exhibited by the wild ecotype. This ecologically driven metabolic divergence reinforces the importance of conserving wild populations as reservoirs of enriched phytochemical profiles with potentially higher therapeutic value.

### 4.3. Cytotoxic Selectivity and Chemotherapeutic Relevance

Cytotoxicity profiling revealed selective potency against human carcinoma lines, with pronounced activity in HepG2 hepatoma cells (IC_50_ = 85.2 ± 3.1 µg/mL) > A549 > MCF-7 (Figure 8). This represents a 2.3-fold enhancement over cultivated ecotypes (HepG2 IC_50_ = 192.4 µg/mL) [50]. The augmented cytotoxicity correlates directly with elevated berberine and palmatine levels—DNA-intercalating isoquinolines that disrupt cell cycle progression in hepatocarcinoma [51,52]. Crucially, the extract maintained an optimal efficacy-safety equilibrium, supporting its preliminary potential as a plant-derived chemoadjuvant. Berberine’s dual targeting of DNA (via topoisomerase inhibition) and inflammation (via NF-κB suppression) highlights a possible synergistic mechanism in hepatocellular carcinoma management that may help overcome drug resistance, though this requires direct experimental validation.

### 4.4. Genetic Polymorphism and Biosynthetic Reprogramming

Genetic analysis uncovered a C → T SNP in the gen1 primer-binding region (Figure 3, panel 1), compromising PCR amplification in wild accessions. While gen2 amplification was unaffected, this mutation may disrupt transcriptional regulation of biosynthetic genes. Such polymorphisms are established drivers of chemotype evolution [53,54,55], often upregulating defense metabolites under ecological stress. Our findings indicate wild *T. crispa* underwent adaptive metabolic reprogramming, evidenced by elevated phytochemical yields and morphological adaptations (leaf curling, stem pigmentation; Figure 4). This SNP could serve as a useful molecular marker for chemotype identification, aiding in the quality assurance of wild-sourced material. It further suggests that stress-optimized cultivation might be a viable strategy to enhance bioactive production, warranting further agronomic research.

### 4.5. Histopathological and Biochemical Safety Profile

Repeated oral dosing (200–600 mg/kg) elicited no detectable hepatorenal toxicity. Biochemical markers (ALT, AST, ALP, urea, creatinine) remained within physiological ranges (Table 8) and showed a trend toward reduction compared to controls. Histopathology confirmed tissue integrity: liver sections showed preserved cord architecture without necrosis or inflammation; kidneys exhibited intact glomerulotubular structures. These findings align with prior subacute toxicity reports [56,57,58] and affirm a preliminary safety profile at the tested doses. The observed dose-dependent reduction in hepatic/renal biomarkers, while requiring validation in disease models, suggests the extract may possess protective properties that could be explored in contexts such as chemotherapy-induced organ injury.

### 4.6. Correlation Between Phytochemicals and Bioactivity

The correlation analysis delineates a hierarchy of biological potency among the phytochemicals, with isoquinoline alkaloids, notably berberine, emerging as the most compelling candidates. Berberine’s consistent efficacy across anti-inflammatory, antioxidant, and cytotoxicity assays underscores its status as a multi-target agent. Its potent cytotoxicity against HepG2 and A549 cells warrants deeper mechanistic investigation. Furthermore, the superior antioxidant capacity of the extracts compared to most pure compounds suggests synergistic interactions within the complex mixture, a phenomenon attributed to complementary radical-scavenging activities. This correlation between potent antioxidant and anti-inflammatory activities reinforces the interconnected nature of oxidative stress and inflammatory pathways. Collectively, these findings prioritize specific compounds like berberine for lead optimization and validate the ethnopharmacological use of polyphenol-rich extracts.

### 4.7. Overall Implications and Future Directions

Collectively, this multidisciplinary investigation establishes wild *T. crispa* as a superior chemotype with enhanced phytochemistry, targeted bioactivities, and a robust preliminary safety profile. Convergent genetic-metabolic-pharmacological evidence underscores its potential viability for development into standardized phytotherapeutics against inflammation and hepatocellular carcinoma. Future research should prioritize controlled cultivation strategies that mimic stress conditions to enhance bioactive yields, chronic toxicity studies to establish long-term safety, and mechanistic investigations to elucidate gene–metabolite interactions.

### 4.8. Limitations and Considerations for Generalizability

While this study provides a comprehensive multidisciplinary profile of *T. crispa*, several limitations should be acknowledged. The plant materials were sourced from two locations within Egypt’s Nile Delta, and thus the specific phytochemical and genetic profile reported here is most representative of this ecotype. Extrapolation to populations from other biogeographical regions should be approached cautiously. Furthermore, the identified SNP, while correlated with metabolic differences, has not been functionally validated; future work employing transcriptomic or genome-wide association studies is needed to establish causality. The 14-day toxicity study provides valuable preliminary safety data but does not assess long-term effects. Finally, the mechanistic insights, while supportive, require deeper molecular validation to fully elucidate the pathways involved in the observed bioactivities.

## 5. Conclusions

This multidisciplinary investigation establishes *Tinospora crispa* (L.) Hook.f. & Thomson as a phytochemically superior candidate within the “Phyllo-stem arboreum” group. Wild ecotypes exhibited significantly higher levels of flavonoids, phenolic acids, tannins, and alkaloids compared to cultivated counterparts, reflecting an adaptive metabolic response to environmental stress. Advanced metabolomic profiling (UHPLC-ESI-QTOF-MS and NMR) confirmed the presence of pharmacologically relevant compounds including berberine, chlorogenic acid, and borapetoside C, with their chemical identities verified through validated MS/MS fragmentation patterns.

Genetic fingerprinting revealed a stress-associated C → T SNP in the gen1 locus, correlating with enhanced secondary metabolite biosynthesis and morphological adaptations, suggesting a potential molecular basis for the observed chemotypic variation. Bioassays demonstrated potent dose-dependent anti-inflammatory activity through NF-κB suppression, alongside selective cytotoxicity against HepG2 hepatocellular carcinoma cells, indicating therapeutic potential in inflammation-associated cancers.

Safety evaluations, including biochemical assays and histopathological analyses, confirmed the absence of detectable hepatorenal toxicity during the 14-day study period at doses up to 600 mg/kg, establishing a preliminary safety profile for short-term use. The trend toward reduced biochemical markers suggests possible protective effects that warrant further investigation.

Collectively, these findings provide scientific validation for the ethnopharmacological uses of *T. crispa* and highlight its potential as a standardized botanical resource. Future research should prioritize controlled cultivation strategies that mimic stress conditions to enhance bioactive yields, chronic toxicity studies to establish long-term safety, and mechanistic investigations to elucidate gene–metabolite interactions. Additionally, functional validation of the identified SNPs and clinical studies are essential to fully realize the translational potential of this resilient, stress-adapted medicinal plant in phytopharmaceutical development.

## Figures and Tables

**Table 1:** Source, Authentication, and Database Reference of Human Cancer Cell Lines Employed in the Study.

Cell Line	ATCC Code	Organism/Origin	Authentication	Database Reference
HepG2	HB-8065	Human hepatocellular carcinoma	STR-profiled; mycoplasma-free	Cellosaurus CVCL_0027 [32]
A549	CCL-185	Human lung carcinoma	STR-profiled; mycoplasma-free	Cellosaurus CVCL_0023 [33]
MCF-7	HTB-22	Human breast adenocarcinoma	STR-profiled; mycoplasma-free	Cellosaurus CVCL_0031 [34,35]

**Table 2:** Quantitative Comparison of Major Phytochemical Screening in Four Climbing Herbaceous Plants Referred to as “Phyllo-stem arboreum”.

Compound Class	*T. crispa*	*T. cordifolia*	*C. pareira*	*S. japonica*	*p*-Value (ANOVA)
Alkaloids	12.50 ± 0.80 a	8.20 ± 0.60 b	6.30 ± 0.45 d	7.80 ± 0.55 c	<0.001
Flavonoids	9.80 ± 0.55 b	15.20 ± 0.90 a	4.50 ± 0.35 c	5.20 ± 0.40 c	<0.001
Phenolic Acids	22.40 ± 1.20 a	9.50 ± 0.75 b	3.80 ± 0.30 c	4.50 ± 0.35 c	<0.001
Saponins	8.20 ± 0.60 a	1.50 ± 0.20 c	6.80 ± 0.50 b	ND	<0.001
Tannins	14.30 ± 0.85 a	12.80 ± 0.75 a	0.90 ± 0.15 b	1.20 ± 0.18 b	<0.001
Terpenoids	7.50 ± 0.55 a	5.80 ± 0.45 b	2.20 ± 0.25 d	3.50 ± 0.30 c	<0.001

Values are presented as mean ± standard deviation (SD; *n* = 3). Different lowercase letters (a–d) within the same row indicate statistically significant differences between species means as determined by one-way ANOVA followed by Tukey’s honestly significant difference (HSD) post hoc test (*p* < 0.05). Means sharing the same letter are not significantly different. The abbreviation “ND” denotes “Not Detected.” This analysis confirms that *T. crispa* possesses a statistically superior phytochemical profile, exhibiting the highest significant content of alkaloids, phenolic acids, and terpenoids among the studied species.

**Table 3:** Morphological Adaptations in Wild *T. crispa* Under Environmental Stress.

Morphological Trait	Reference (Herbarium/Cultivated, Panels 2–3)	Field-Collected Sample (Panel 1)	Observed Change
Leaf shape and size	Broad, fully cordate leaves with smooth margins	Smaller leaves, slightly lobed with undulating margins	Leaf area reduction and marginal waviness under stress
Leaf texture and color	Thin, uniformly deep green lamina	Thicker, somewhat tougher lamina, green but less lustrous	Slights chlorophyll and reduced apparent chlorophyll density
Stem architecture and surface	Relatively straight, uniformly cylindrical green stems with fine warts	Arched, more irregular stems with pronounced nodal swellings and rough, prickly surface	Increased stem lignification and armature for mechanical protection
Fruit surface and shape	Plump, ovoid drupes with smoother surface (panel 2)	More elongated, pendant ovoid fruits with clearer longitudinal ridges (panel 1)	Shape elongation and accentuated ribbing associated with stressful habitat

**Table 4:** Phytochemical Comparison Between Wild and Reference *T. crispa* (Mean ± SD).

Parameter	Wild *T. crispa*	Ref *T. crispa*	% Change	*p*-Value (*t*-test)
Total Flavonoids (mg QE/g)	17.5 ± 1.6 a	12.2 ± 1.3 b	+43.4%	<0.01
Total Polyphenols (mg GAE/g)	28.1 ± 2.1 a	19.6 ± 1.7 b	+43.4%	<0.01
Tannins (mg CE/g)	7.9 ± 0.6 a	4.8 ± 0.4 b	+64.6%	<0.001
Total Chlorophyll (mg/g FW)	1.32 ± 0.09 b	3.05 ± 0.11 a	−56.7%	<0.001

Note: Different superscript letters (a, b) within the same row indicate a statistically significant difference between wild and reference ecotypes as determined by an unpaired, two-tailed Student’s *t*-test (*p* < 0.05).

**Table 5:** Quantitative LC–MS Profiling of Major Bioactive Metabolites Identified in Wild *T. crispa* Extract.

Peak #	RT (Min)	*m/z*	Molecular Formula	Ion Type	MS^2^ Fragments (*m/z*)	Putative Identification	Concentration in Wild *T. crispa* (mg/g DW, Mean ± SD)	Significance Letter (Wild vs. Cultivated)
1	8.3	355.103	C_16_H_18_O_9_	[M + H]^+^	163.04, 135.03	Chlorogenic acid	22.4 ± 1.2	^a^
2	9.1	611.161	C_27_H_30_O_16_	[M + H]^+^	303.05, 271.02, 151.00	Rutin	4.6 ± 0.4	^a^
3	12.92	336.123	C_20_H_18_NO_4_^+^	[M]^+^	321.09, 306.07, 292.06	Berberine	12.5 ± 0.8	^a^
4	15.35	352.134	C_21_H_22_NO_4_^+^	[M]^+^	337.12, 320.10, 292.08	Palmatine	6.2 ± 0.5	^a/b^
5	19.54	342.17	C_18_H_23_NO_5_^+^	[M]^+^	251.14, 131.08	Magnoflorine	3.1 ± 0.3	^a^
6	20.35	571.192	C_28_H_35_O_12_	[M + H]^+^	409.15, 247.10, 108.05, 230.13, 262.28	Borapetoside C	7.8 ± 0.6	

Values are expressed as mean ± SD (*n* = 3). Different superscript letters within a row indicate significant differences between wild and cultivated ecotypes at *p* < 0.05 (one-way ANOVA followed by Tukey’s post hoc test). Natural range “max” values are taken from previously reported phytochemical surveys of *Tinospora* spp. and related *Menispermaceae* taxa.

**Table 6:** Compilation of Key ^1^H-NMR Signals for Selected Natural Compounds.

Compound	δ (^1^H) ppm	Multiplicity	Integration	Key Proton Assignment
Chlorogenic acid	7.58	d (J = 15.9 Hz)	1H	H-7′ (trans-cinnamoyl)
	6.28	d (J = 15.9 Hz)	1H	H-8′
Rutin	12.61	s	1H	5-OH (chelated)
	7.68	d (J = 2.0 Hz)	1H	H-2′ (flavone)
Berberine	9.89	s	1H	H-8 (isoquinoline)
	6.18	s	2H	-OCH_2_O- (dioxymethylene)
Palmatine	9.85	s	1H	H-8
	4.05	s	3H	-OCH_3_ (methoxy)
Magnoflorine	6.78	s	1H	H-1 (aporphine)
	3.45	s	6H	-N^+^(CH_3_)_2_ (dimethylamino)
Borapetoside C	5.58	dd (J = 3.5,12.0)	1H	H-3 (olefinic)
	4.92	d (J = 12.0 Hz)	1H	H-17a (exomethylene)
	4.75	d (J = 12.0 Hz)	1H	H-17b

**Table 7:** Cytotoxicity profiles and comparative statistical analysis of *T. crispa* extracts.

Parameter	Wild Extract IC_50_ (µg/mL)	Cultivated Extract IC_50_ (µg/mL)	*p*-Value (*t*-test)	Fold Potency Increase	Doxorubicin (+ Control) IC_50_ (µg/mL)	*p*-Value vs. Dox
HepG2	85.2 ± 3.1 a	142.4 ± 8.5 b	<0.001	1.67×	<2.0	<0.001
A549	132.7 ± 5.4 a	175.0 ± 9.2 b	<0.001	1.32×	<2.0	<0.001
MCF-7	158.3 ± 6.8 a	200.0 ± 10.1 b	<0.001	1.26×	<2.0	<0.001

Statistical Notes: Different letters (a, b) within the same row indicate statistically significant differences between wild and cultivated extracts (unpaired Student’s *t*-test, *p* < 0.05). Fold Potency Increase = IC_50_ (cultivated)/IC_50_ (wild). For comparisons across cell lines: One-way ANOVA revealed significant differences in sensitivity among cell lines (*p* < 0.001), with HepG2 being the most sensitive and MCF-7 the least sensitive to both extracts. Concentration–response analysis: One-way ANOVA confirmed significant concentration-dependent effects for all cell lines (*p* < 0.001).

**Table 8:** Dose–Response Assessment of Liver (ALT, AST, ALP) and Kidney (Urea, Creatinine) Function Markers.

	Liver Function			Kidney Function	
Group	ALT (U/L)	AST (U/L)	ALP (U/L)	Urea (mg/dL)	Creatinine (mg/dL)
Control	42.1 ± 1.5 a	68.5 ± 2.8 a	110.2 ± 3.5 a	36.5 ± 1.2 a	0.75 ± 0.03 a
Low Dose (200 mg/kg)	40.3 ± 1.3 ab	65.2 ± 2.5 ab	105.3 ± 3.2 ab	35.1 ± 1.1 ab	0.72 ± 0.03 ab
Moderate Dose (400 mg/kg)	38.5 ± 1.4 bc	62.8 ± 2.6 bc	101.5 ± 3.3 bc	34.6 ± 1.0 b	0.69 ± 0.02 b
High Dose (600 mg/kg)	37.2 ± 1.2 c	61.0 ± 2.3 c	98.7 ± 3.0 c	33.8 ± 1.1 b	0.67 ± 0.02 b
ANOVA *p*-value	*p* < 0.01	*p* < 0.01	*p* < 0.01	*p* < 0.05	*p* < 0.05

Data presented as mean ± SD (*n* = 6 per group). Different lowercase letters (a, b, c) within the same column indicate statistically significant differences between groups as determined by one-way ANOVA followed by Tukey’s post hoc test (*p* < 0.05). Values sharing the same letter are not significantly different. Normal physiological ranges: ALT: 30–65 U/L; AST: 50–100 U/L; ALP: 100–300 U/L; Urea: 20–50 mg/dL; Creatinine: 0.2–0.8 mg/dL.

## Data Availability

Data supporting the findings (raw metabolomic, genetic, and histopathological data) are available upon reasonable request from the corresponding author.

## Abbreviations

The following abbreviations are used in this manuscript:

*T. crispa*	*Tinospora crispa* (L.) *Hook.f. & Thomson*
*T. dactyloides*	*Tinospora dactyloides*
*T. cordifolia*	*Tinospora cordifolia*
ARC	Agricultural Research Center
NO	Nitric oxide
NMR	Nuclear magnetic resonance
